# Marek's Disease Virus-Encoded MicroRNA 155 Ortholog Critical for the Induction of Lymphomas Is Not Essential for the Proliferation of Transformed Cell Lines

**DOI:** 10.1128/JVI.00713-19

**Published:** 2019-08-13

**Authors:** Yaoyao Zhang, Na Tang, Jun Luo, Man Teng, Katy Moffat, Zhiqiang Shen, Mick Watson, Venugopal Nair, Yongxiu Yao

**Affiliations:** a The Pirbright Institute & UK-China Centre of Excellence for Research on Avian Diseases, Pirbright, Guildford, Surrey, United Kingdom; b School of Animal Science and Technology, Guangxi University, Nanning, China; c Binzhou Animal Science and Veterinary Medicine Academy & UK-China Centre of Excellence for Research on Avian Diseases, Binzhou, China; d Key Laboratory of Animal Immunology of the Ministry of Agriculture, Henan Provincial Key Laboratory of Animal Immunology, Henan Academy of Agricultural Sciences, Zhengzhou, China; e College of Animal Science and Technology, Henan University of Science and Technology, Luoyang, China; f The Roslin Institute, R(D)SVS, University of Edinburgh, Midlothian, United Kingdom; g The Jenner Institute Laboratories, University of Oxford, Oxford, United Kingdom; h Department of Zoology, University of Oxford, Oxford, United Kingdom; University of Southern California

**Keywords:** CRISPR/Cas9 editing, Marek's disease virus, microRNA, oncogenesis, transformation

## Abstract

MicroRNAs (miRNAs) are small noncoding RNAs with profound regulatory roles in many areas of biology, including cancer. MicroRNA 155 (miR-155), one of the extensively studied multifunctional miRNAs, is important in several human malignancies such as diffuse large B cell lymphoma and chronic lymphocytic leukemia. Moreover, miR-155 orthologs KSHV-miR-K12-11 and MDV-miR-M4, encoded by Kaposi’s sarcoma-associated herpesvirus (KSHV) and Marek’s disease virus (MDV), respectively, are also involved in oncogenesis. In MDV-induced T-cell lymphomas and in lymphoblastoid cell lines derived from them, MDV-miR-M4 is highly expressed. Using excellent disease models of infection in natural avian hosts, we showed previously that MDV-miR-M4 is critical for the induction of T-cell lymphomas as mutant viruses with precise deletions were significantly compromised in their oncogenicity. However, those studies did not elucidate whether continued expression of MDV-miR-M4 is essential for maintaining the transformed phenotype of tumor cells. Here using an *in situ* CRISPR/Cas9 editing approach, we deleted MDV-miR-M4 from the MDV-induced lymphoma-derived lymphoblastoid cell line MDCC-HP8. Precise deletion of MDV-miR-M4 was confirmed by PCR, sequencing, quantitative reverse transcription-PCR (qRT-PCR), and functional analysis. Continued proliferation of the MDV-miR-M4-deleted cell lines demonstrated that MDV-miR-M4 expression is not essential for maintaining the transformed phenotype, despite its initial critical role in the induction of lymphomas. Ability to examine the direct role of oncogenic miRNAs *in situ* in tumor cell lines is valuable in delineating distinct determinants and pathways associated with the induction or maintenance of transformation in cancer cells and will also contribute significantly to gaining further insights into the biology of oncogenic herpesviruses.

**IMPORTANCE** Marek’s disease virus (MDV) is an alphaherpesvirus associated with Marek’s disease (MD), a highly contagious neoplastic disease of chickens. MD serves as an excellent model for studying virus-induced T-cell lymphomas in the natural chicken hosts. Among the limited set of genes associated with MD oncogenicity, MDV-miR-M4, a highly expressed viral ortholog of the oncogenic miR-155, has received extensive attention due to its direct role in the induction of lymphomas. Using a targeted CRISPR-Cas9-based gene editing approach in MDV-transformed lymphoblastoid cell lines, we show that MDV-miR-M4, despite its critical role in the induction of tumors, is not essential for maintaining the transformed phenotype and continuous proliferation. As far as we know, this was the first study in which precise editing of an oncogenic miRNA was carried out *in situ* in MD lymphoma-derived cell lines to demonstrate that it is not essential in maintaining the transformed phenotype.

## Abstract (precis)

Marek’s disease virus (MDV) is an alphaherpesvirus associated with Marek’s disease (MD), a highly contagious neoplastic disease of chickens. MD serves as an excellent model for studying virus-induced T-cell lymphomas in the natural chicken hosts. Among the limited set of genes associated with MD oncogenicity, MDV-miR-M4, a highly expressed viral ortholog of the oncogenic miR-155, has received extensive attention due to its direct role in the induction of lymphomas. Using a targeted CRISPR-Cas9-based gene editing approach in MDV-transformed lymphoblastoid cell lines, we show that MDV-miR-M4, despite its critical role in the induction of tumors, is not essential for maintaining the transformed phenotype and continuous proliferation. As far as we know, this was the first study in which precise editing of an oncogenic miRNA was carried out *in situ* in MD lymphoma-derived cell lines to demonstrate that it is not essential in maintaining the transformed phenotype.

## INTRODUCTION

MicroRNAs (miRNAs) are ∼22-nucleotide (nt) small RNA molecules that function as master regulators of gene expression in many species, including plants, worms, flies, and animals, as well as in a number of viruses. Most of the virus-encoded miRNAs are seen in DNA viruses, with members of the family *Herpesviridae* accounting for the vast majority demonstrating the significance of miRNA-mediated gene regulation in the biology of herpesvirus infection (1–3). Identification of miRNAs encoded by human oncogenic gammaherpesviruses such as Kaposi’s sarcoma-associated herpesvirus (KSHV) and Epstein-Barr virus (EBV) as well as avian oncogenic alphaherpesvirus Marek’s disease virus (MDV) has highlighted the potential contribution of the virus-encoded miRNAs to the oncogenicity of these viruses. Among the several roles of the herpesvirus-encoded miRNAs, such as immune evasion and control of viral latency/lytic replication and oncogenic potential (4–6), the role of viral orthologs of host microRNA 155 (miR-155) encoded by KSHV and MDV in oncogenesis has been the most extensively studied (5, 7). As a multifunctional miRNA expressed primarily in the hematopoietic cells and in cells of the immune systems, microRNA 155 (miR-155) is highly conserved in most species, including humans and chickens, and is associated with different lymphomas (8–11). In EBV-induced B-cell transformation as well as in a number of EBV-associated B-cell lymphomas, including Hodgkin’s lymphoma, diffuse large B-cell lymphoma (DLBCL), and Burkitt’s lymphoma in humans, upregulation of miR-155 resulting in escalated cell proliferation and neoplastic transformation has been reported (12, 13). KSHV, a human gammaherpesvirus associated with lymphoproliferative disorders such as primary effusion lymphoma (PEL), multicentric Castleman disease (MCD), and B lymphomagenesis in AIDS patients, encodes 25 miRNAs. Among these miRNAs, KSHV-K12-11, which plays critical role in pathogenesis, is a functional ortholog of hsa-miR-155 sharing identical seed sequences (14–16). MDV encodes MDV-miR-M4-5p (miR-M4), a functional ortholog with seed sequences identical to those of miR-155 and KSHV-K12-11 that has been shown to play a critical role in the induction of lymphomas (6).

Marek’s disease (MD) is a lymphoproliferative disease of chickens characterized by rapid-onset lymphomas in multiple organs and by infiltration into peripheral nerves, causing paralysis. MD serves as an excellent model for studying virus-induced T-cell lymphomas. Among the more than 100 genes carried by the MDV (17, 18), the gene encoding the basic leucine zipper protein Meq (MDV EcoRI Q), which is undisputedly expressed in both lytic and latent infections, is the most important viral gene associated with MD oncogenicity (19, 20). Deletion of the Meq gene or inhibition of its important interactions with host proteins such as c-Jun, c-Fos, and C-terminal binding protein (CtBP) can affect the oncogenicity of the virus (21–23). Although the viral telomerase RNA (vTR) also has been shown to promote MDV-induced oncogenesis (24), the role of MDV-encoded miRNAs in oncogenesis has drawn extensive attention (25–27). MDV encodes 14 miRNA precursors producing 26 mature miRNAs which are clustered into three separate genomic loci within the repeat regions of the viral genome. MDV-miR-M4, located in cluster 1, was shown previously to be the viral ortholog of miR-155 (28). The oncogenic properties of miR-155, together with the observation of a high level of miR-M4 expression in tumor cells and the identification of several cancer pathway-related target genes, suggested the important role of this miRNA in MDV-induced oncogenesis. Indeed, we and others have previously demonstrated the direct role of miR-M4 in the induction of tumors using recombinant MDV engineered to have deletion mutations or seed region mutations in miR-M4 by the use of *in vivo* experiments in chickens (6, 29). Furthermore, we showed previously that the loss of the oncogenic phenotype of a miR-M4 deletion mutant of MDV can be partially rescued by MDV expressing gga-miR-155, demonstrating the similarities in the functions of the two orthologs (6). While the role of miR-M4 in the induction of MD lymphomas has been clearly demonstrated in these studies, it remains unclear whether continued high-level expression of miR-M4 is essential for maintaining the transformed phenotype of MDV-transformed tumor cells. As clonal populations of transformed tumor cells with latent MDV genome and limited gene expression (30–32), lymphoblastoid cell lines (LCL) derived from MD lymphomas have served as valuable resources to improve understanding of distinct aspects of virus-host interactions in transformed cells. However, detailed investigations into the role of different viral and host determinants in these cells have been difficult due to the lack of tools for manipulation of viral/host genomes of these cells *in situ*. Following our recent success in efficient editing of the MDV genome in cell culture systems that support lytic virus replication *in vitro* (33), we explored the use of a gene editing approach in an MDCC-HP8 cell line that is latently infected with the GA strain of MDV. Using MDCC-HP8 cells that stably expressed Cas9 and synthetic guide RNAs (gRNAs) with a two-part guide RNA system, we examined the effect of deletion of miR-M4 to gain insights into its functional role. Continued proliferation of the miR-M4 knockout cell lines suggested that expression of miR-M4 gene is not essential for maintenance of the transformed state of the MDCC-HP8 tumor cell line, despite its known critical role in the induction of MD lymphomas.

## RESULTS

### Knockout of MDV-miR-M4 in HP8 cells.

**FIG 1:**
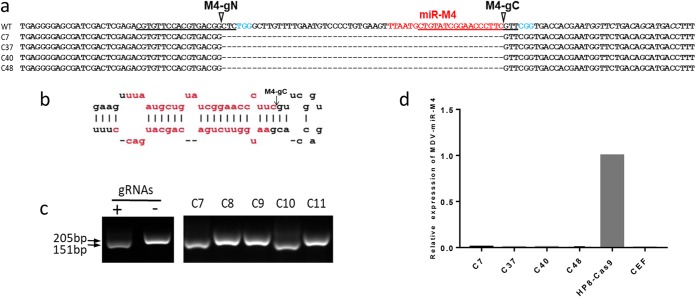
Deletion of miR-M4 by CRISPR/Cas9 editing in HP8 cells. (a) Nucleic acid sequences of the truncated/edited PCR products showing the successful deletion of miR-M4 on selected clones. The target sequence is underlined, the PAM sequence is indicated in light blue, and the cleavage site is indicated by an arrow. (b) The predicted stem-loop structure of the pre-miR-M4, with the predicted cleavage site indicated by an arrow. The mature miRNA sequences are shown in red. (c) PCR amplification of the edited region using primers miR-M4-F and miR-M4-R on the cell lysates of transfected cells at 2 days posttransfection and on isolated single-cell clones C7 to C11. (d) Relative expression levels of miR-M4, measured by qRT-PCR in RNA extracted from miR-M4-deleted clones C7, C37, C40, and C48 along with unedited HP8-Cas9 and CEF. The value corresponding to the level of miR-M4 in HP8-Cas9 was set as 1 for calibration.

On the basis of our success in efficient editing of the MDV genome during lytic replication in infected chicken embryo fibroblast (CEF) cultures in our previous studies (33), we attempted editing of the latent MDV genome in virus-transformed cell lines. The initial attempt performed with a transfected gRNA-Cas9 expression plasmid showed low editing efficiency, thought to be largely due to the relatively low transfection efficiency of the hard-to-transfect MDV-transformed cell lines (data not shown). A new gene editing strategy involving the transfection of synthetic gRNAs with a two-part guide RNA system into a MDV-transformed cell line stably expressing Cas9 (HP8-Cas9) showed great success. For the targeted editing of MDV-miR-M4 in the latent viral genome in this cell line, two gRNAs, M4-gN and M4-gC, were designed using CRISPR guide RNA designing software (http://crispr.mit.edu/). M4-gN targeted the upstream sequence of the mature miR-M4 sequence, and M4-gC targeted the sequence spanning the mature miR-M4 sequence and the loop region of the pre-miRNA hairpin structure, resulting in the predicted cleavage site lying exactly at the end of the miR-M4 mature sequence (Fig. 1a and b). Successful miR-M4 deletion would release a 54-nt fragment following the successful cleavage of the sequence by the two gRNAs. Considering the presence of several MDV genomes integrated in multiple chromosomes of the chicken genome determined on the basis of fluorescence *in situ* hybridization (FISH) analysis (unpublished data) and the location of miR-M4 in the terminal repeat region, which doubles the number of copies of miR-M4, two distinct bands are expected to be identified by the use of PCR tests on the genomic DNA from cells harvested 48 h after transfection and the use of specific primers located at the flanking region of Cas9 targeting sites. The top band of around 205 bp represented the unedited sequence or edited target site(s), with small indels present if the two sites were not cleaved simultaneously. The bottom, smaller band product of around 151 bp corresponded to the edited region with a 54-bp deletion between the two Cas9 cleavage sites. Interestingly, only the bottom band was detected by PCR analysis, indicating the highly efficient cleavage resulting from the use of the two gRNAs, with the majority of the cells transfected and edited efficiently. Despite the observation of only a single band, single-cell sorting was carried out to obtain a pure population of miR-M4-deleted cell. Although only the bottom band was obtained by PCR before sorting was performed in the mixed population, clones with the top band were predominant after single-cell cloning (Fig. 1c). Sequence analysis of four bottom bands confirmed that the results represented the direct end joining (EJ) product of two predicted Cas9 target sites (Fig. 1a). Interestingly, the sequences of all four clones were identical, suggesting that further screening of several additional clones might be required to identify variations within the edited sequences. The successful knockout of the miR-M4 sequence was further confirmed by reverse transcription-quantitative PCR (qRT-PCR) analysis, using uninfected CEFs as a negative control. As expected, miR-M4 was absent from all four miR-M4-deleted HP8 clones and control CEF, contrasting with the high-level expression detected in the parental HP8-Cas9 cells (Fig. 1d). These experiments demonstrated that miR-M4 had been deleted successfully by the use of the two-part guide RNA system in the HP8 cell line stably expressing Cas9.

### miR-M4 is not essential in maintaining the transformed phenotype of MDV-transformed cell line.

**FIG 2:**
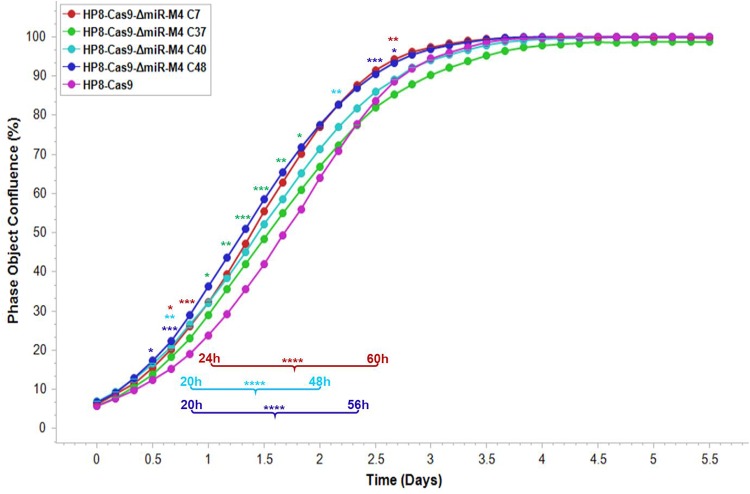
Proliferation of the HP8-Cas9 and the miR-M4-deleted clones monitored in real time using IncuCyte S3 live imaging system. The cell phase object confluence of each cell population was determined every 4 h for 132 h from 4 separate regions per well and 4 wells per sample in 96-well plates by IncuCyte and compared with that determined from the HP8-Cas9 control. Growth curves are shown as means ± standard errors (SE) representative of results from three independent experiments. Asterisks (*) indicate statistically significant differences between miR-M4-deleted clones and parental HP8-Cas9 cells at different times. *, *P* < 0.05; **, *P* < 0.01; ***, *P* < 0.001; ****, *P* < 0.0001. Asterisks are placed above the time points (single time points) or underneath the growth curves for those time points with the same results during the indicated period of time.

miR-M4 has been shown to be essential for the MDV in inducing tumors (6, 29). To explore the role of miR-M4 in maintaining the transformed state, we examined the effect of deletion of miR-M4 on the proliferation of HP8 cells. For this, we carried out kinetic monitoring of proliferation of the wild-type HP8-Cas9 strain and the miR-M4-deleted clones using an IncuCyte S3 live-cell imaging system (Fig. 2). The cell proliferation data obtained in real time from the images collected at 4-h intervals showed that the miR-M4-deleted clones proliferated at a significantly higher rate within the first 3 days than the parental HP8-Cas9 cells, although different clones showed different levels of significance at various time points. These results suggested that expression of miR-M4 was not essential for the proliferation phenotype of these transformed cells.

### Pu.1 is upregulated in HP8-ΔmiR-M4 cells.

**FIG 3:**
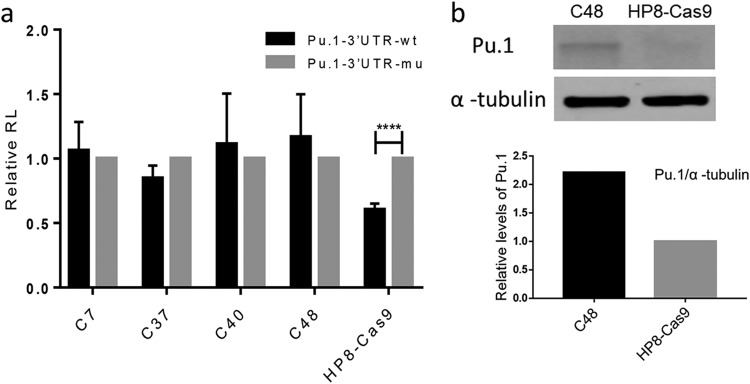
Successful deletion of miR-M4 measured by functional studies. (a) Firefly and *Renilla* luciferase (RL) activities were measured consecutively with the dual luciferase reporter system (Promega) following transfection of reporter constructs containing the wild-type (wt) or mutant (mu) MRE region of the 3′ UTR of miR-M4 target gene Pu.1 into the miR-M4-deleted cells and the parental HP8-Cas9. The relative expression levels of *Renilla* luciferase were determined with the normalized levels of firefly luciferase. For each sample, values from four replicates representative of results from at least two independent experiments were used in the analysis. The value determined for the psiCHECK-2 mutant was set as 1. Error bars are derived from four replicates. (b) Immunoprecipitation (IP)-Western blot analysis of Pu.1 in miR-M4-deleted HP8 clone C48 and HP8-Cas9. Matched inputs were assayed for α-tubulin as loading control. The relative signal intensities of the Pu.1 Western blot band were quantified using ImageQuant and normalized against the corresponding signal from the tubulin band. The value corresponding to the signal from HP8-Cas9 cells was set as 1.

Having shown that miR-M4 can be deleted from HP8 cell line and that it is not essential for the continued proliferation of the transformed cells, we wanted to examine the effect of miR-M4 deletion on expression of its target proteins. For this, we chose to analyze the expression levels of Pu.1, one of the very well characterized and validated miR-M4 targets (28). The expression levels were first assessed using a luciferase reporter assay by transfection of the reporter construct containing the wild-type predicted miR-M4 response element (MRE) or the mutant MRE region of the 3′ untranslated region (UTR) of Pu.1 into the miR-M4-deleted and the parental HP8-Cas9 cells. This assay showed that the relative *Renilla* luciferase levels of reporter constructs with wild-type MRE sequences were reduced by nearly 40 % compared with the levels seen with the mutant MRE construct in the parental HP8-Cas9 cells. Compared to this, such a reduction of luciferase levels was absent in all of the miR-M4-deleted clones (Fig. 3a), demonstrating the functional effect of miR-M4 deletion on the Pu.1 target. Next, we determined the miR-M4-mediated silencing by directly measuring the level of Pu.1 expression in one of the selected mutant clones, i.e., clone C48, along with the parental cells. Immunoprecipitation-Western blot analysis showed that the Pu.1 expression level was much higher in the miR-M4-deleted cells than in the parental cells (Fig. 3b). Results from the reporter assay and direct expression analysis of the Pu.1 target thus confirmed the deletion of miR-M4 and the functional consequences in the mutant C48 clone.

### Effect of miR-M4 deletion on expression of other viral miRNAs and Meq protein.

**FIG 4:**
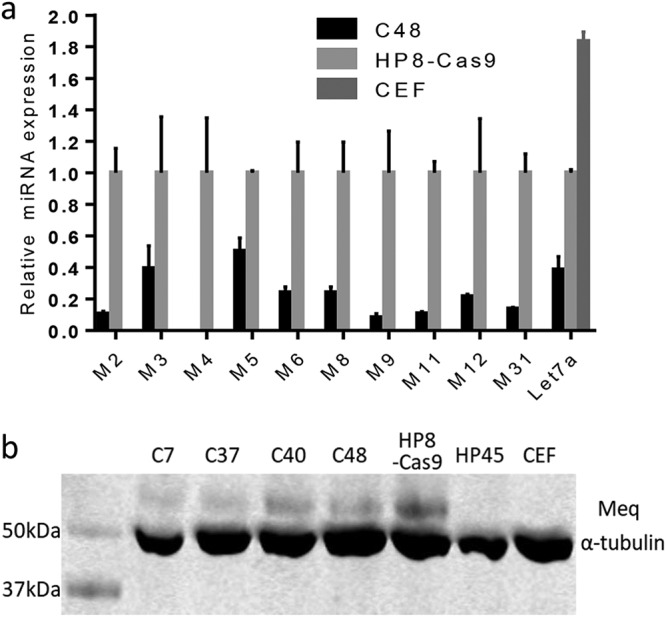
MDV miRNA and Meq protein expression in miR-M4-deleted cells. (a) Relative expression levels of the indicated viral miRNAs and host miRNA let-7a were measured by qRT-PCR with RNA extracted from miR-M4-deleted clone C48 along with the unedited HP8-Cas9 and CEF. All values were normalized to the level of expression of the endogenous GAPDH gene, and levels were calculated as fold expression change relative to those from CEF. The value corresponding to the level of each miRNA in HP8-Cs9 was set as 1. (b) Detection of Meq expression by Western blotting with anti-Meq monoclonal antibody FD7 in HP8-Cas9 and HP8-Cas9-ΔmiR-M4 clones. ALV-transformed B-cell line HP45 and uninfected CEF were included as negative controls. For the loading control, the same blot was stripped and reprobed with anti-α-tubulin antibody.

Having demonstrated successful knockout of miR-M4 from the MDV genome in the HP8 cell line, we next analyzed the effect of miR-M4 deletion on expression of other MDV-encoded miRNAs and the Meq major viral oncoprotein. The 14 MDV-encoded miRNA precursors are clustered into three separate genomic loci. Cluster 1 (the Meq cluster), containing miR-M2, miR-M3, miR-M4, miR-M5, miR-M9, and miR-M12, is located upstream of the Meq gene. The midcluster, containing three miRNA precursors (miR-M11, miR-M31, and miR-M1) is located downstream of Meq. The third cluster, referred to as the LAT cluster, lies within the first intron of the latency-associated transcript (LAT). To assess the potential effect of miR-M4 deletion on other miRNAs, we first amplified the cluster 1 miRNAs by PCR with the primers at the flanking region of the cluster. The sequence of the PCR product was determined to confirm the absence of any changes (data not shown) except for the edited region as shown in Fig. 1a. Next, we analyzed the level of expression of each miRNA in cluster 1, miR-M31 from cluster 2, and miR-M6 and miR-M8 from cluster 3 by the use of the RNA extracted from miR-M4-deleted clone 48 and the parental HP8-Cas9. The level of expression of the host miRNA gga-let-7a was also measured, with total RNA from uninfected CEF used as the control. As shown in Fig. 4a, all viral miRNAs were absent and only let-7a was detectable in the CEF sample. Except for the absence of miR-M4 from miR-M4-deleted clone 48, both the viral and the host miRNAs were detected in HP8 before or after miR-M4 deletion. Quantitation of selected viral miRNAs by qRT-PCR indicated that they were still expressed in miR-M4-deleted clone 48, although their expression levels differed from those seen with the parental HP8 cells (Fig. 4a). We also examined Meq expression in the miR-M4-deleted cells by Western blot analysis. Avian leukosis virus (ALV)-transformed B-cell line HP45 and uninfected CEFs which do not express Meq were used as negative controls. Results of the Western blot analysis confirmed the expression of Meq in the miR-M4-deleted cells, demonstrating that miR-M4 was not required for Meq expression in these cells (Fig. 4b).

### v-*rel* relieves the inhibition of miR-155 expression in HP8-ΔmiR-M4.

**FIG 5:**
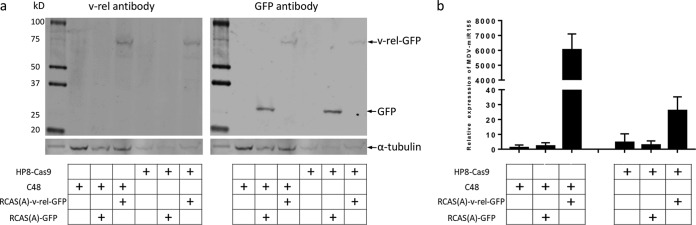
Upregulation of miR-155 in miR-M4-deleted HP8 by v-*rel*. (a) Detection of v-*rel* expression with anti-v-*rel* monoclonal antibody HY87 and GFP expression with anti-GFP antibody by Western blotting in HP8-ΔmiR-M4 clone C48 and HP8-Cas9 infected with RCAS(A)-GFP and RCAS(A)-v-*rel*-GFP, respectively. For the loading control, the same blot was stripped and reprobed with anti-α-tubulin antibody. (b) Relative levels of miR-155 expression were detected by qRT-PCR in HP8-ΔmiR-M4 clone C48 and HP8-Cas9 infected with RCAS(A)-GFP and RCAS(A)-v-*rel*-GFP, respectively.

We have previously shown that miR-155 is consistently downregulated in MDV-transformed tumors and cell lines (34) and that this downregulation can be rescued by expressing v-*rel*, which also results in activation of the expression of miR-M4 in these cells (35). We wanted to examine whether the downregulation of miR-155 can be rescued without the activation of miR-M4 by transduction of v-*rel* with RCAS(A)-v-*rel*-GFP [RCAS(A)-v-*rel*-green fluorescent protein] virus in HP8-ΔmiR-M4 clone 48. The GFP marker allowed sorting of the RCAS-infected cells. Analysis of the sorted cells by Western blotting confirmed the expression of v-*rel*-GFP in RCAS(A)-v-*rel*-GFP-infected cells and GFP expression in RCAS(A)-GFP infected cells (Fig. 5a). Expression of v-*rel* increased the level of miR-155 expression by approximately 6,026-fold in HP8-ΔmiR-M4 cells but only 25-fold in HP8-Cas8 cells (Fig. 5b), demonstrating that deletion of miR-M4 increased miR-155 expression induced by v-*rel*.

## DISCUSSION

Virus-host interactions in herpesviruses are characterized by long-term survival as latent infections in different cell types. With total dependence on the host cell, several viruses have adopted strategies to modulate the host cellular environment, including the modulation of miRNAs. A number of studies have demonstrated the role of miRNAs in replication, pathogenesis, and oncogenesis of herpesviruses (3, 4, 7, 36–38). These included our own studies demonstrating the critical role of miR-M4 in the induction of lymphomas by MDV (6). While these observations have also been confirmed by other studies (29), the role of viral miRNAs in maintaining the transformed state, as well as in other functions such as the switch of latency/lytic replication in tumor cells, has not been examined. In particular, the role of miR-M4, the viral ortholog of oncogenic miR-155 encoded by oncogenic MDV, in maintaining the transformed phenotype of the tumor cell line is unknown. MDV-transformed LCLs derived from MD lymphomas which contain multiple copies of the MDV genome integrated in different chromosomes are valuable to study latency, transformation, and reactivation *in situ*. Having established the CRISPR/Cas9-based editing of the viral genome at relatively high efficiency in MDV-transformed cell lines, we report here the precise knockout of miR-M4 from the MDV genome in LCL HP8. Results from these studies show that miR-M4, despite its critical role in the induction of lymphomas by oncogenic MDV strains, is not required for the continued proliferation of MDV-transformed HP8 LCL. As far as we know, this was the first study to have made use of the CRISPR/Cas9-based gene editing technology *in situ* to demonstrate that a critical virus-encoded miRNA is not essential to maintain the transformed phenotype of a virus-induced cancer cell line.

By transfection of two forms of synthetic gRNA into HP8 cells stably expressing Cas9, we have shown here that miR-M4 can be deleted at a relatively high level of efficiency (Fig. 1c). Considering the presence of the multiple copies of the target loci in these cell lines, the high editing efficiency highlighted that efficient gRNA, rather than the copy numbers of the target genes, is the key to achieving the desired editing even in hard-to-transfect cell lines such as MDV-transformed LCL. Although the editing efficiency seen with the transfected cell lines appeared to be very high on the basis of the PCR test results, sorting of the single-cell populations did identify a number of unedited clones, further highlighting the importance of single-cell sorting in gene editing pipelines. These findings are also consistent with our observation that the rate of recovery of edited cells is probably much lower than that of the unedited cell populations, suggesting that single-cell cloning is a required step to get the pure populations of the edited cells regardless of the efficiency of gene editing. The successful knockout of miR-M4 demonstrated the value of this approach in identifying other molecular determinants associated with different phenotypes, including the latency/lytic switch in LCLs. While the growth of the miR-M4-deleted cells confirmed that miR-M4 expression is not essential for maintenance of the transformation and proliferation of LCL, the ΔmiR-M4 cell line that we have generated will also be a valuable research tool in the future for addressing significant biological issues concerning the functional role of this important miRNA homolog. For example, it will be interesting to learn if the populations of shared target genes of MDV-miR-M4, miR-155, and KSHV-miR-K12-11 (5) are upregulated in miR-M4-deleted cells and downregulated after the v-*rel* transduction which activates miR-155 expression (Fig. 5b). Similarly, future studies employing global analysis of the changes in the transcriptome and proteomes of the edited cell populations, together with analysis of the changes in the viral and host epigenomes, will provide more insights into the fine tuning of the molecular regulatory network around the members of these families of miRNAs in these virus-transformed cell lines. Finally, these cells also provide the opportunity to investigate the role of miR-M4 in induction of lymphomas (transplantable tumors) *in vivo* in experimentally infected target chicken hosts.

Repair by nonhomologous end joining (NHEJ) is usually accompanied by random nucleotide insertions/deletions at the cleavage site. As a result, the edited sequence is likely to represent a mixed population. However, sequencing results have shown that virtually all of the edited sequences are end joining products of the two predicted Cas9 cleavage sites. Although additional variations may be discovered when more clones are analyzed, the edited loci often contained only the predominant mutant sequences, as we have shown previously (33, 39). The reasons for the clonal nature of the appearance of the single population are not fully clear. Whether this is related to the stable expression of Cas9 in these cells or to other factors requires further investigation.

The oncogene v-*rel* activates miR-155 expression by binding to NF-κB site in a Bic promoter. We have shown previously that the downregulation of miR-155 in MDV-transformed cell lines could be rescued by expressing v-*rel* in these cells (35). Using the same approach, we have shown here that the downregulation of miR-155 can also be rescued in the context of miR-M4-deleted HP8 by transduction of v-*rel* with RCAS(A)-v-*rel*-GFP virus in HP8-ΔmiR-M4 clone C48. Interestingly, only a 25-fold increase in the miR-155 level could be induced in the unedited HP8-Cas9 cells compared to a 6,026-fold increase in miR-M4-deleted clone C48, suggesting that the absence of miR-M4 significantly enhances the ability of v-*rel* to induce miR-155 expression in MDV tumor cell lines. As has been demonstrated previously, miR-M4 is highly expressed in MDV tumor cell lines compared to miR-155, which is actively downregulated, although the precise mechanisms of the differential downregulation have not been identified. On the basis of the findings from the present study, it appears that the downregulation of miR-155 may be directly linked to the high level of miR-M4 expression, as the activation of miR-155 by v-*rel* was more robust in the miR-M4-deleted cells. However, further studies are required to delineate the associated mechanisms involved in such regulation.

The precise editing of the miR-M4 locus to abolish the expression of mature miR-M4 in MDV-induced T-lymphoma-derived cell line HP8 clearly demonstrated that the proliferative capacity of the transformed cell line is not dependent on continued high-level expression of miR-M4. The continued proliferation of cells is unlikely to be due to the inability to express other viral miRNAs such as all other miRNAs in cluster 1 and selected miRNAs from both the midcluster and LAT cluster detected by miRNA qRT-PCR (Fig. 4a). MDV-miR-M4 is very important for the oncogenicity of MDV, but other miRNAs in the cluster also contribute, since the mutant virus expressing miR-M4 alone in cluster 1 remained nononcogenic (6). Whether or not other versions of miRNA contribute to the maintenance of transformed phenotype remains to be elucidated. The continued proliferation of cells is also not due to the lack of expression of adjacent viral gene such as Meq, as we were able to demonstrate expression of the protein by Western blot analysis (Fig. 4b). The significantly increased proliferation capacity of miR-M4 knockout clones suggests that miR-M4 in these contexts may have a proliferation suppressor function. Additional studies on the detailed analysis of the gene expression profiles of these clones will be required to gain further insights into the biology of miR-M4 in these cells. Although it is possible that LCLs may have acquired other mutations that may have made them no longer dependent on miR-M4 for proliferation, the failure of attempts to rescue the Meq-deleted cell line after repeated attempts indicated that this is unlikely to be the case. Whether or not other genes or miRNAs are involved in maintaining the transformed phenotype of MD tumor cell lines remains to be investigated.

## MATERIALS AND METHODS

### Cell culture.

The MDV-transformed HP8 lymphoblastoid cell lines (40) from a GA strain-induced tumor were grown at 38.5°C in 5% CO_2_ in RPMI 1640 medium (Life technologies) containing 10% fetal bovine serum, 10% tryptose phosphate broth, 1% sodium pyruvate solution (Sigma), and 100 units/ml of penicillin and streptomycin (Life Technologies).

### gRNAs.

A two-part guide RNA system containing a crRNA:tracrRNA guide complex was used for editing. The sequences of gRNA miR-M4-gN and miR-M4-gC (listed in Table 1) were used for production of synthetic crRNAs by Integrated DNA Technologies (IDT, USA). The tracrRNA was purchased from IDT. The lyophilized crRNA and tracrRNA pellets were resuspended in Duplex buffer (IDT) at a 200 μM concentration and stored in small aliquots at −80°C.

**TABLE 1:** List of primer sequences

Primer	Sequence (5′–3′)
miR-M4-gN	CGTGTTCCACGTGACGGCTC
miR-M4-gC	CTGTATCGGAACCCTTCGTT
miR-M4-F	TGAGGGGAGCGATCGACTC
miR-M4-R	GATTCAATATTACATCACTTCAACGG

### Generation and characterization of HP8-ΔmiR-M4 cell line.

An NEPA21 electroporator was used for the transfection of HP8 cells that stably expressed Cas9 (HP8-Cas9) (41). For deletion of miR-M4, 1 × 10^6^ of HP8-Cas9 cells were resuspended in 96 μl Opti-MEM medium. Two crRNAs (miR-M4-gN and miR-M4-gC) were mixed with equal molar amounts of tracrRNA to reach a final duplex concentration of 100 μM in 4 μl of duplex buffer and incubated at 95°C for 5 min. After the duplex was allowed to cool to room temperature, it was mixed with cell suspension and electroporated under conditions of 275 V and 1.5-ms pulse width of poring pulse. At 48 h postelectroporation, 1 × 10^5^ cells were harvested and analyzed by PCR. The remaining cells were sorted into 96 wells for single-cell isolation. After 7 days of incubation, cells were collected and analyzed by PCR. The cells harvested for PCR analysis were lysed in 1× protein K-based DNA isolation buffer (33) at 65°C for 30 min. A 1-μl volume of extracted DNA template was used for PCR with primers outside the targeted sites to identify the correct miR-M4 gene knockout. The primer sequences of miR-M4-F and miR-M4-R used for PCR are listed in Table 1.

### RCAS virus infection.

Virus stocks were generated from DF-1 cells transfected with RCAS(A)-EGFP [RCAS(A)-enhanced GFP] and RCAS(A)-v-*rel*-EGFP constructs approximately 5 days after transfection, when 100% of the cells were EGFP positive. For v-*rel* transduction in HP8-Cas9 and HP8-ΔmiR-M4, 1 ml (∼10^6^ 50% tissue culture infective doses [TCID_50_]) of RCAS(A)-EGFP or RCAS(A)-v-*rel*-EGFP virus stock was used to infect 1 × 10^6^ HP8-ΔmiR-M4 cells and 1 × 10^6^ HP8-Cas9 cells. EGFP-expressing RCAS(A)-v-*rel*-EGFP-infected and RCAS(A)-EGFP-infected HP8-ΔmiR-M4 and HP8-Cas9 cells were sorted into 6-well plates. After 7 days of incubation, cells were collected and examined for v-*rel* and EGFP expression by Western blotting and for miR-155 expression by qRT-PCR.

### Sorting.

For single-cell cloning, cells were washed twice with phosphate-buffered saline (PBS) containing 5% fetal bovine serum (FBS) and centrifuged at 450 × *g* for 5 min at room temperature. The cell pellets were resuspended in cold PBS–5% FBS and sorted into 96-well U-bottom plates (Corning) containing growth medium by fluorescence-activated cell sorter (FACS) analysis using a FACSAria II system (BD Bioscience).

### qRT-PCR analysis of miRNA expression.

The expression levels of miRNAs were analyzed using a TaqMan MicroRNA assay system (Life Technologies) and 10 ng total RNA as a template for reverse transcription. Each reverse transcription reaction was tested by PCR in triplicate and performed twice independently. For relative quantification of miRNA-M4 in HP8-ΔmiR-M4 cells (Fig. 1d) and of miR-155 in v-*rel*-transduced cells (Fig. 5b), all values were normalized to the expression level of endogenous let-7a, and levels were calculated as fold expression change relative to those from HP8-Cas9 cells (miR-155) and CEFs (miR-M4). For relative quantification of viral miRNAs and host gga-let-7a in HP8-ΔmiR-M4 clone 48 and controls HP8-Cas9 and CEF (Fig. 4a), all values were normalized to the level of expression of the endogenous GAPDH (glyceraldehyde-3-phosphate dehydrogenase) gene, and levels were calculated as fold expression change relative to those from CEF.

### Dual luciferase reporter assay.

A previously constructed reporter construct for the validated miR-M4 and miR-155 target Pu.1 in psiCHECK vector was used to measure the miR-M4 activity in HP8 (28). The reporter constructs contain a 110-bp fragment of the chicken Pu.1 3′ untranslated region (UTR) sequence with MRE wild-type sequence (Pu.1-3′UTR-wt) or MRE mutant sequence (Pu.1-3′UTR-mu) inserted downstream of *Renilla* luciferase in the psiCHECK-2 vector (Promega) (28). HP8-ΔmiR-M4 cells and HP8-Cas9 cells (5 × 10^5^) were transfected with 4 μg of either Pu.1-3′UTR-wt or Pu.1-3′UTR-mu using an NEPA21 electroporator as described above. The luciferase expression was assayed 48 h later using a Dual-Glo luciferase assay system (Promega) and following the manufacturer’s instructions. The relative levels of expression of *Renilla* luciferase were determined with the normalized levels of firefly luciferase. For each sample, values from four replicates representative of results from at least two independent experiments were used in the analysis.

### Western blotting.

Approximately 1 × 10^6^ HP8-ΔmiR-M4 cells and the control cells were collected and boiled with TruPAGE LDS sample buffer (Sigma) for 10 min. The samples were separated on a 4% to 12% TruPAGE precast gel, and the resolved proteins were transferred onto polyvinylidene difluoride (PVDF) membranes. Expression levels of Meq, Pu.1, v-*rel*, and GFP were detected using anti-Meq monoclonal antibody (MAb) FD7 (21), rabbit anti-SPIB polyclonal antibody (Aviva Systems Biology), anti-v-*rel* MAb HY87 (35), and GFP polyclonal antibody (SICGEN), respectively. The loading control used in all cases was α-tubulin (Sigma-Aldrich). After probing with primary antibodies was performed, the blots were incubated with secondary antibody IRDye®680RD goat anti-mouse IgG (Li-Cor) (for detection of Meq, v-*rel*, and α-tubulin), IRDye®800CW donkey anti-rabbit IgG (Li-Cor) (for detection of Pu.1), and IRDye®800CW donkey anti-goat IgG (Li-Cor) (for detection of GFP), and the results were visualized using Odyssey Clx (Li-Cor). For GFP detection, the PVDF membrane used for v-*rel* detection was stripped and reprobed with GFP antibody following the same procedure.

### Analysis of HP8-Cas9-ΔmiR-M4 cell growth.

The growth of HP8-Cas9-ΔmiR-M4 clones along with unedited HP8-Cas9 was monitored by IncuCyte S3 live-cell imaging (Essen Bioscience Ltd., Hertfordshire, United Kingdom). Briefly, 8,000 cells were seeded in a 96-well plate (Corning) and images were captured every 4 h for 132 h from four separate regions per well using a 10× objective. By recording the phase object confluence, the levels of growth of the HP8-Cas9-ΔmiR-M4 clones were compared with that of parental HP8-Cas9. IncuCyte data were analyzed by two-way analysis of variance (ANOVA) with Tukey’s multiple-comparison test using GraphPad Prism version 7.01 (GraphPad Software, Inc., San Diego, CA). The results are shown as means ± standard errors (SE) of results from four replicates each with 4 separate regions per well representative of three independent experiments. *P* values of <0.05 were considered to be significant.
